# Aqua­{4,4′-dibromo-6,6′-dimeth­oxy-2,2′-[ethane-1,2-diylbis(nitrilo­methyl­idyne)]diphenolato}copper(II)

**DOI:** 10.1107/S1600536809046212

**Published:** 2009-11-14

**Authors:** Hai Xie

**Affiliations:** a College of Chemistry & Chemical Engineering, Shanxi Datong University, Shanxi 037009, People’s Republic of China

## Abstract

The title complex, [Cu(C_18_H_16_Br_2_N_2_O_4_)(H_2_O)], lies on a crystallographic mirror plane with the Cu^II^ ion coordinated by two N atoms and two O atoms of a tetra­dentate Schiff base ligand and one O atom from a water ligand in a slightly distorted square-pyramidal environment. The mirror plane, which coincides with the Cu—O_water_ bond, imposes disorder of the atoms of the ethyl­ene group. In the crystal structure, inter­molecular O—H⋯O hydrogen bonds link complex mol­ecules into extended chains along [100].

## Related literature

For related structures, see: Nathan *et al.* (2003 ▶); Saha *et al.* (2007 ▶); Xing (2009 ▶). For general background to Schiff base compounds, see: Yu *et al.* (2007 ▶); Ghosh *et al.* (2006 ▶); Singh *et al.* (2007 ▶); Nayka *et al.* (2006 ▶).
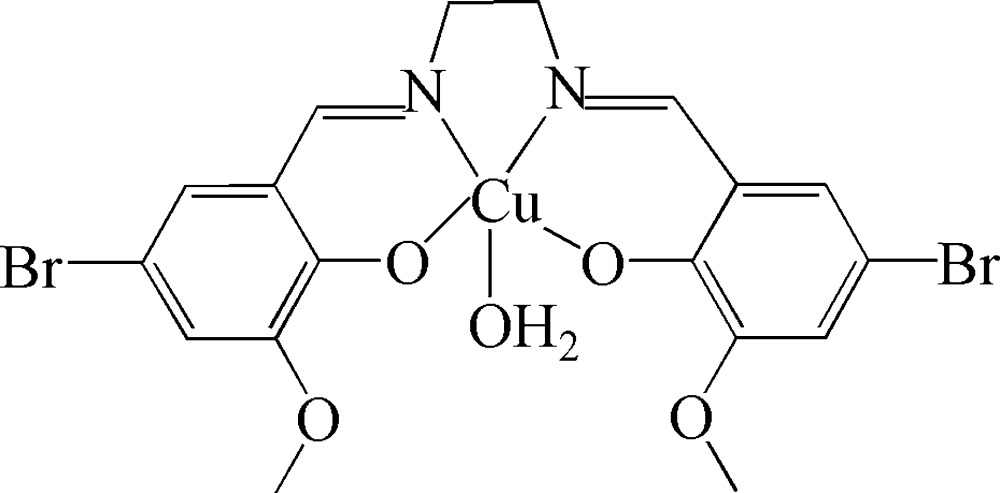


## Experimental

### 

#### Crystal data

[Cu(C_18_H_16_Br_2_N_2_O_4_)(H_2_O)]
*M* *_r_* = 565.70
Orthorhombic, 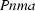

*a* = 8.7299 (13) Å
*b* = 27.968 (4) Å
*c* = 7.9900 (12) Å
*V* = 1950.8 (5) Å^3^
*Z* = 4
Mo *K*α radiation
μ = 5.25 mm^−1^
*T* = 293 K
0.23 × 0.20 × 0.18 mm

#### Data collection

Bruker APEXII CCD diffractometer
Absorption correction: multi-scan (*SADABS*; Sheldrick, 1996 ▶) *T*_min_ = 0.378, *T*_max_ = 0.452
8970 measured reflections
1759 independent reflections
1498 reflections with *I* > 2σ(*I*)
*R*_int_ = 0.034

#### Refinement

*R*[*F* ^2^ > 2σ(*F* ^2^)] = 0.053
*wR*(*F* ^2^) = 0.138
*S* = 1.13
1759 reflections
140 parameters
1 restraint
H-atom parameters constrained
Δρ_max_ = 1.48 e Å^−3^
Δρ_min_ = −1.02 e Å^−3^

### 

Data collection: *APEX2* (Bruker, 2004 ▶); cell refinement: *SAINT-Plus* (Bruker, 2001 ▶); data reduction: *SAINT-Plus*; program(s) used to solve structure: *SHELXS97* (Sheldrick, 2008 ▶); program(s) used to refine structure: *SHELXL97* (Sheldrick, 2008 ▶); molecular graphics: *SHELXTL* (Sheldrick, 2008 ▶); software used to prepare material for publication: *SHELXTL*.

## Supplementary Material

Crystal structure: contains datablocks I, global. DOI: 10.1107/S1600536809046212/lh2938sup1.cif

Structure factors: contains datablocks I. DOI: 10.1107/S1600536809046212/lh2938Isup2.hkl

Additional supplementary materials:  crystallographic information; 3D view; checkCIF report

## Figures and Tables

**Table 1:** Hydrogen-bond geometry (Å, °)

*D*—H⋯*A*	*D*—H	H⋯*A*	*D*⋯*A*	*D*—H⋯*A*
O3—H3*A*⋯O2^i^	0.82	2.23	2.963 (5)	150
O3—H3*A*⋯O1^i^	0.82	2.27	2.936 (7)	139

Symmetry code: (i) 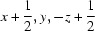.

**Fig. 1.**
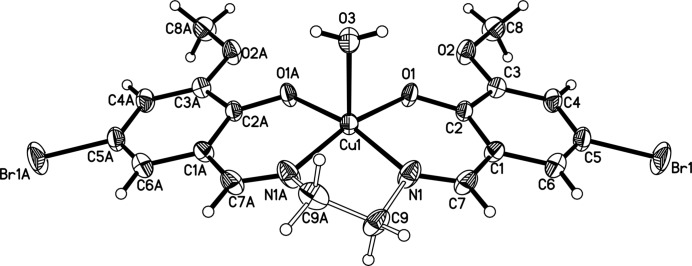
The molecular structure of the title compound with the atom-labelling scheme. Displacement ellipsoids are drawn at the 30% probability level. All H-atoms are omitted for clarity. Only one disorder component is shown with open bonds [symmetry code (A): (x, -y+1/2, z).

## supplementary crystallographic information

### Comment

Schiff-bases can readily form stable complexes with most transition metals, in which some may exhibit interesting properties (Yu *et al.*, 2007; Ghosh *et al.*, 2006; Singh *et al.*, 2007; Nayka *et al.*, 2006). Here, we report a Cu(II) complex based on the tetradentate Schiff-base ligand *N*,*N*'-ethylenebis(5-bromo-3-methoxysalicylaldimine.

The molecular structure of the title compound is shown in Fig. 1. The complex lies on a crystallographic mirror plane with the Cu^II^ ion coordinated in a slightly distorted square-pyramidal environment. The basal plane is occupied by two N atoms and two O atoms of the Schiff-base ligand, and the apical site is occupied by the O atom of the coordinated water molecule. The Cu^II^ ion is displaced towards the Cu—O_water_ bond from the plane formed by the two N atoms and two O atoms by 0.224 (4) Å. The Cu—N and Cu—O bond lengths are consistent with the corresponding distances found in other Cu Schiff base complexes (Nathan, *et al.*, 2003; Saha, *et al.*, 2007; Xing, 2009).

### Experimental

Condensation of ethyl diamine and 5-bromo-3-methoxyl-2-hydroxy-benzaldehyde with the ratio 1:2 in ethanol gave the Schiff base ligand. The title compound was synthesized by treatment Cu(ClO_4_)_2_.6H_2_O and the schiff-base ligand (1:1, molar ratio) in methanol. After the mixture was stirred for for about 30 min at room temperature, it was filtered and the filtrate was allowed to partial evaporate in air for one week to produce crystals suitable for X-ray diffraction with a yield about 52%.

### Refinement

H atoms were included using the HFIX command in *SHELXL-97* (Sheldrick, 2008), with C—H = 0.96 and 0.93 Å; O—H = 0.82Å and were allowed for as riding atoms with *U*_iso_(H) = 1.5*U*_eq_(C_methyl_) and (*U*_iso_(H) = 1.2*U*_eq_(C,*O*).

### Crystal data

**Table:**

[Cu(C_18_H_16_Br_2_N_2_O_4_)(H_2_O)]	*F*(000) = 1116
*M**_r_* = 565.70	*D*_x_ = 1.926 Mg m^−^^3^
Orthorhombic, *P**n**m**a*	Mo *K*α radiation, λ = 0.71073 Å
Hall symbol: -P 2ac 2n	Cell parameters from 2580 reflections
*a* = 8.7299 (13) Å	θ = 2.9–26.7°
*b* = 27.968 (4) Å	µ = 5.25 mm^−^^1^
*c* = 7.9900 (12) Å	*T* = 293 K
*V* = 1950.8 (5) Å^3^	Block, blue
*Z* = 4	0.23 × 0.20 × 0.18 mm

### Data collection

**Table:**

Bruker APEXII CCD diffractometer	1759 independent reflections
Radiation source: fine-focus sealed tube	1498 reflections with *I* > 2σ(*I*)
graphite	*R*_int_ = 0.034
φ and ω scans	θ_max_ = 25.0°, θ_min_ = 2.7°
Absorption correction: multi-scan (*SADABS*; Sheldrick, 1996)	*h* = −7→10
*T*_min_ = 0.378, *T*_max_ = 0.452	*k* = −33→27
8970 measured reflections	*l* = −8→9

### Refinement

**Table:**

Refinement on *F*^2^	Primary atom site location: structure-invariant direct methods
Least-squares matrix: full	Secondary atom site location: difference Fourier map
*R*[*F*^2^ > 2σ(*F*^2^)] = 0.053	Hydrogen site location: inferred from neighbouring sites
*wR*(*F*^2^) = 0.138	H-atom parameters constrained
*S* = 1.13	*w* = 1/[σ^2^(*F*_o_^2^) + (0.0394*P*)^2^ + 13.8845*P*] where *P* = (*F*_o_^2^ + 2*F*_c_^2^)/3
1759 reflections	(Δ/σ)_max_ = 0.001
140 parameters	Δρ_max_ = 1.48 e Å^−^^3^
1 restraint	Δρ_min_ = −1.01 e Å^−^^3^

### Special details

**Table:**

Geometry. All esds (except the esd in the dihedral angle between two l.s. planes) are estimated using the full covariance matrix. The cell esds are taken into account individually in the estimation of esds in distances, angles and torsion angles; correlations between esds in cell parameters are only used when they are defined by crystal symmetry. An approximate (isotropic) treatment of cell esds is used for estimating esds involving l.s. planes.
Refinement. Refinement of F^2^ against ALL reflections. The weighted R-factor wR and goodness of fit S are based on F^2^, conventional R-factors R are based on F, with F set to zero for negative F^2^. The threshold expression of F^2^ > 2sigma(F^2^) is used only for calculating R-factors(gt) etc. and is not relevant to the choice of reflections for refinement. R-factors based on F^2^ are statistically about twice as large as those based on F, and R- factors based on ALL data will be even larger.

### Fractional atomic coordinates and isotropic or equivalent isotropic displacement parameters (Å^2^)

**Table:**

	*x*	*y*	*z*	*U*_iso_*/*U*_eq_	Occ. (<1)
Br1	0.28985 (13)	0.50680 (3)	−0.14560 (14)	0.0802 (4)	
Cu1	0.43677 (12)	0.2500	0.01791 (14)	0.0336 (3)	
O1	0.2831 (5)	0.29928 (14)	0.0459 (6)	0.0394 (11)	
O2	0.0393 (5)	0.35017 (16)	0.0910 (7)	0.0492 (12)	
O3	0.5275 (7)	0.2500	0.2889 (8)	0.0446 (16)	
H3A	0.5656	0.2746	0.3257	0.067*	
N1	0.5856 (6)	0.2962 (2)	−0.0675 (8)	0.0515 (16)	
C1	0.4226 (7)	0.3656 (2)	−0.0697 (8)	0.0385 (15)	
C2	0.2929 (7)	0.3439 (2)	0.0005 (8)	0.0334 (13)	
C3	0.1626 (7)	0.3736 (2)	0.0235 (8)	0.0387 (15)	
C4	0.1622 (8)	0.4211 (2)	−0.0169 (9)	0.0436 (16)	
H4	0.0754	0.4397	0.0006	0.052*	
C5	0.2941 (9)	0.4410 (2)	−0.0848 (9)	0.0472 (17)	
C6	0.4200 (9)	0.4150 (2)	−0.1124 (9)	0.0484 (18)	
H6	0.5062	0.4291	−0.1596	0.058*	
C7	0.5621 (8)	0.3402 (2)	−0.1013 (9)	0.0460 (17)	
H7	0.6421	0.3571	−0.1505	0.055*	
C8	−0.0958 (9)	0.3769 (3)	0.1200 (10)	0.058 (2)	
H8A	−0.0753	0.4015	0.2011	0.087*	
H8B	−0.1746	0.3561	0.1615	0.087*	
H8C	−0.1290	0.3913	0.0172	0.087*	
C9	0.7175 (16)	0.2701 (7)	−0.147 (2)	0.054 (5)	0.50
H9A	0.6948	0.2643	−0.2645	0.065*	0.50
H9B	0.8089	0.2897	−0.1413	0.065*	0.50
C9A	0.7457 (13)	0.2236 (7)	−0.061 (3)	0.052 (5)	0.50
H9A1	0.7812	0.2280	0.0527	0.063*	0.50
H9A2	0.8177	0.2037	−0.1222	0.063*	0.50

### Atomic displacement parameters (Å^2^)

**Table:**

	*U*^11^	*U*^22^	*U*^33^	*U*^12^	*U*^13^	*U*^23^
Br1	0.1002 (8)	0.0319 (4)	0.1084 (8)	0.0054 (4)	0.0121 (6)	0.0259 (4)
Cu1	0.0301 (5)	0.0275 (5)	0.0433 (6)	0.000	0.0040 (5)	0.000
O1	0.038 (2)	0.024 (2)	0.056 (3)	0.0025 (18)	0.006 (2)	0.010 (2)
O2	0.038 (3)	0.034 (2)	0.076 (3)	0.006 (2)	0.014 (2)	0.007 (2)
O3	0.053 (4)	0.032 (3)	0.049 (4)	0.000	−0.011 (3)	0.000
N1	0.035 (3)	0.044 (3)	0.076 (4)	0.000 (3)	0.013 (3)	0.020 (3)
C1	0.041 (4)	0.037 (4)	0.038 (3)	−0.002 (3)	0.001 (3)	0.008 (3)
C2	0.039 (3)	0.026 (3)	0.035 (3)	0.000 (3)	0.000 (3)	0.002 (3)
C3	0.041 (3)	0.035 (3)	0.040 (3)	−0.002 (3)	−0.001 (3)	0.002 (3)
C4	0.049 (4)	0.030 (3)	0.051 (4)	0.005 (3)	−0.004 (3)	0.007 (3)
C5	0.065 (5)	0.029 (3)	0.048 (4)	−0.001 (3)	−0.005 (4)	0.005 (3)
C6	0.054 (4)	0.039 (4)	0.052 (4)	−0.004 (3)	0.000 (4)	0.009 (3)
C7	0.038 (4)	0.043 (4)	0.057 (4)	−0.005 (3)	0.008 (3)	0.013 (3)
C8	0.052 (5)	0.053 (5)	0.069 (5)	0.015 (4)	0.011 (4)	0.009 (4)
C9	0.031 (8)	0.053 (9)	0.078 (14)	−0.001 (7)	0.009 (9)	0.021 (10)
C9A	0.023 (8)	0.067 (11)	0.066 (13)	0.004 (7)	−0.006 (8)	−0.011 (11)

### Geometric parameters (Å, °)

**Table:**

Br1—C5	1.903 (7)	C2—C3	1.420 (9)
Cu1—O1^i^	1.937 (4)	C3—C4	1.367 (9)
Cu1—O1	1.937 (4)	C4—C5	1.389 (10)
Cu1—N1	1.954 (5)	C4—H4	0.9300
Cu1—N1^i^	1.954 (5)	C5—C6	1.338 (10)
Cu1—O3	2.305 (6)	C6—H6	0.9300
O1—C2	1.303 (7)	C7—H7	0.9300
O2—C3	1.371 (8)	C8—H8A	0.9600
O2—C8	1.416 (8)	C8—H8B	0.9600
O3—H3A	0.8188	C8—H8C	0.9600
N1—C7	1.277 (9)	C9—C9A	1.491 (19)
N1—C9A^i^	1.504 (10)	C9—H9A	0.9700
N1—C9	1.505 (10)	C9—H9B	0.9700
C1—C2	1.402 (9)	C9A—N1^i^	1.504 (10)
C1—C6	1.421 (9)	C9A—H9A1	0.9700
C1—C7	1.433 (10)	C9A—H9A2	0.9700
			
O1^i^—Cu1—O1	90.8 (2)	C5—C4—H4	120.6
O1^i^—Cu1—N1	166.2 (3)	C6—C5—C4	121.8 (6)
O1—Cu1—N1	91.8 (2)	C6—C5—Br1	120.1 (6)
O1^i^—Cu1—N1^i^	91.8 (2)	C4—C5—Br1	118.1 (5)
O1—Cu1—N1^i^	166.2 (3)	C5—C6—C1	120.2 (7)
N1—Cu1—N1^i^	82.7 (4)	C5—C6—H6	119.9
O1^i^—Cu1—O3	97.45 (18)	C1—C6—H6	119.9
O1—Cu1—O3	97.45 (18)	N1—C7—C1	125.3 (6)
N1—Cu1—O3	95.7 (2)	N1—C7—H7	117.4
N1^i^—Cu1—O3	95.7 (2)	C1—C7—H7	117.4
C2—O1—Cu1	127.2 (4)	O2—C8—H8A	109.5
C3—O2—C8	117.8 (5)	O2—C8—H8B	109.5
Cu1—O3—H3A	118.5	H8A—C8—H8B	109.5
C7—N1—C9A^i^	120.7 (10)	O2—C8—H8C	109.5
C7—N1—C9	120.0 (9)	H8A—C8—H8C	109.5
C7—N1—Cu1	127.2 (5)	H8B—C8—H8C	109.5
C9A^i^—N1—Cu1	111.3 (9)	C9A—C9—N1	110.7 (14)
C9—N1—Cu1	109.7 (8)	C9A—C9—H9A	109.5
C2—C1—C6	120.2 (6)	N1—C9—H9A	109.5
C2—C1—C7	122.8 (6)	C9A—C9—H9B	109.5
C6—C1—C7	117.0 (6)	N1—C9—H9B	109.5
O1—C2—C1	125.4 (6)	H9A—C9—H9B	108.1
O1—C2—C3	118.1 (6)	C9—C9A—N1^i^	98.7 (12)
C1—C2—C3	116.5 (6)	C9—C9A—H9A1	112.0
C4—C3—O2	123.7 (6)	N1^i^—C9A—H9A1	112.0
C4—C3—C2	122.6 (6)	C9—C9A—H9A2	112.0
O2—C3—C2	113.7 (5)	N1^i^—C9A—H9A2	112.0
C3—C4—C5	118.7 (7)	H9A1—C9A—H9A2	109.7
C3—C4—H4	120.6		

Symmetry codes: (i) *x*, −*y*+1/2, *z*.

### Hydrogen-bond geometry (Å, °)

**Table:**

*D*—H···*A*	*D*—H	H···*A*	*D*···*A*	*D*—H···*A*
O3—H3A···O2^ii^	0.82	2.23	2.963 (5)	150
O3—H3A···O1^ii^	0.82	2.27	2.936 (7)	139

Symmetry codes: (ii) *x*+1/2, *y*, −*z*+1/2.
